# Teamwork for eye care

**Published:** 2014

**Authors:** M Babar Qureshi

**Affiliations:** Director: Neglected Tropical Diseases, CBM and Chair: ICO committee on training teams, Cambridge, UK. mbqureshi1@gmail.com

**Figure:**
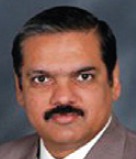
M Babar Qureshi

Human resource development (HRD) – the development of the people who deliver health care – has been identified as one of the key pillars of eye health delivery. HRD is one of the essential building blocks of the World Health Organization (WHO) Global Action Plan: ‘Towards universal eye health’. The importance of HRD is also recognised beyond eye care, as can be seen in the WHO Health Systems approach.

Historically, eye care delivery was mainly the responsibility of ophthalmologists. It soon became clear, however, that in order to effectively reduce avoidable blindness, other types of health care workers would need to be developed, trained and deployed to work with and support ophthalmologists. A team approach would therefore be essential.

In recent years, eye care team development has become an important part of the advocacy and action plans of most global eye health agencies and regional bodies. The International Agency for the Prevention of Blindness (IAPB) and the International Council of Ophthalmology (ICO) both have international committees on HRD, and IAPB has also formed regional HRD committees. One of their key tasks has been to identify gaps and plan HRD for individual groups of eye health providers – including ophthalmic nurses, ophthalmic clinical officers, and optometrists/refractionists – in a way that supports the development of the eye care team as a whole.

**Figure:**
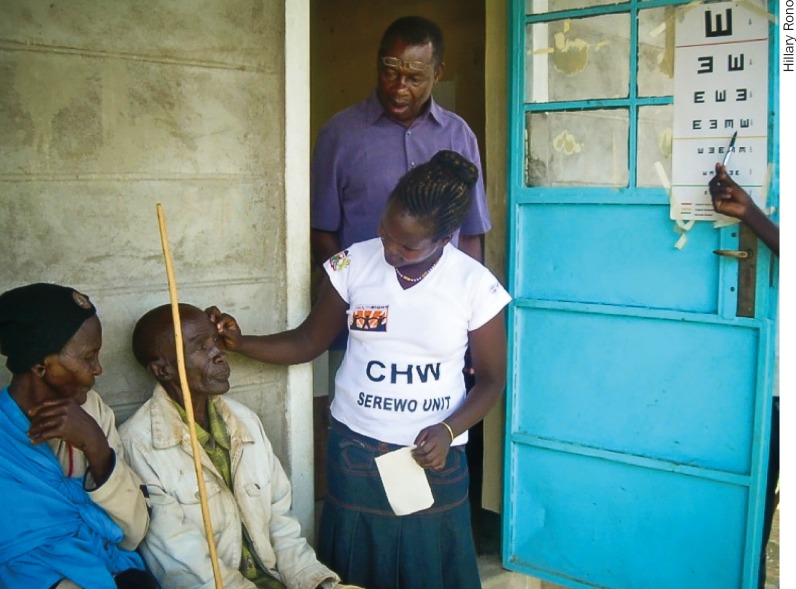
A ophthalmic clinical officer observes a community health worker examining a patient's eyes so that he can offer helpful feedback and support, if needed

The composition of an eye care team varies from region to region and country to country, and it will also differ depending on whether the team is working in a national eye care programme or in a rural eye clinic. The goal is the same, however: to provide high quality eye care to the satisfaction of the patient.

**ABOUT THIS ISSUE**

**Figure:**
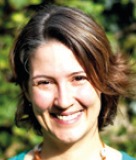
Elmien Wolvaardt Ellison

Editor: *Community Eye Health Journal.*

This issue is about teamwork for eye care, and about how eye teams can function more effectively to address blindness and improve eye health in the communities where the need is greatest.

Our authors look at the various people involved: ophthalmic nurses, ophthalmic clinical officers, ophthalmologists, optometrists, the surgical team, and managers. But there are many more people whose enthusiastic and skilled participation is essential for both good clinical outcomes and a good patient experience: low vision and rehabilitation workers, teachers, community health workers, equipment technicians, receptionists, cleaners, drivers, and outreach workers – to name but a few.

A good indicator of a team that is functioning well is that people are enjoying working together. What can be better than doing worthwhile work with people you respect and appreciate, and who each bring unique skills, perspectives and energy to the team?

But great teamwork does not always come naturally. This issue therefore looks at the importance of establishing a culture of teamwork right at the start, when people are undergoing training. We focus on good leadership, which is needed to keep team members focused on what is important, on the goals the team wants to achieve, and on good planning and management, all of which helps to support and facilitate teamwork. There is advice on how to function within a team and improve the effectiveness of the team. Other articles look at various groups of eye health workers, the challenges they face, and how these are being addressed to improve teamwork.

We hope you will enjoy this issue and that you will be inspired to make positive changes where you work.

## Leadership: a crucial component of teamwork

Teams need leaders who are knowledgeable, skilled, highly motivated, and who aim to offer high quality, sustainable eye care services. A good leader will consult with the team, take their opinions on board, and create a vision and goals that are genuinely shared by everyone; this means that the team members will feel personally motivated to achieve these shared goals. Good leaders are able to champion the team's vision and goals with energy and enthusiasm, inspiring their team members to keep going during difficult times. She or he will share credit and be able to manage the negative fall-out if things go wrong.

The team leader sets the tone for the team and is responsible for maintaining the team's values and the team culture’, i.e. what behaviour is acceptable and what is not. The team leader can and should model the correct attitudes and behaviour for the rest of the team. For example:

- showing professional respect for the skills and limitations of each member of the eye team
- demonstrating professional dilligence
- being willing to listen to others
- making decisions based on a collective and evidence-based approach (rather than for personal gain)
- treating people fairly, whatever their cultural background, gender, sexual orientation or health/disability.

By being fair, available, and communicative, the team leader can ensure that there is good communication among the rest of the team and that working relationships are positive.

Leaders also have a facilitative role: ensuring that the team has everything they need to achieve their goals, such as appropriate (and functioning) equipment, a reliable supply of medicines and consumables, good systems and protocols, and a clean and safe environment in which to work. Managers (see page 30) can support the team leader in this role.

A good leader also keeps the professional growth of the team in mind by proactively seeking career development for the team and ensuring that everyone has a clear job description, detailing their roles and responsibilities. Team members should understand where their job fits into the health system and what their opportunities for career progression are.

## Being a good team member

The team needs to be able to support the leader in achieving high performance and quality of service, delivered to the satisfaction of the patients they serve. Team members must be professionally competent in their field of work, diligent and committed, and should view their leaders as mentors and facilitators.

It makes sense to develop the attitudes and skills needed for successful teamwork from the outset, during training (see page 28). A curriculum geared towards teamwork will encourage and train eye care workers to:

- identify themselves as team members
- communicate clearly and respectfully
- develop critical thinking and problem solving skills
- make decisions and respect the decisions of others
- support and complement the work of others
- trust other members of the team
- give clear and prompt feedback
- motivate themselves and others
- keep learning.

These ideas and skills should be included in the curriculum and taught both explicitly (during course work) and implicitly (through the example set by leaders during practical training). Changing the curriculum is always a challenge, but this can be overcome through advocacy and sharing succesful examples of a curriculum focused on the team.

## Task shifting

To enhance the quantity and quality of an eye service, task shifting has been used with a lot of success. Task shifting means delegation of tasks within the team to complement one another. This can take place informally (at the level of the institution) or formally (i.e. at ministry of health level, with the creation of a new role such as that of cataract surgeon).

One of the best examples of task shifting has been the use of mid-level personnel, optometrists and nurses to undertake many of the tasks which the ophthalmologist used to do in the past, thereby giving the ophthalmologist more time for specialised tasks or surgery that only she or he is qualified to perform. Mid-level eye care workers are also task shifting their previous activities to community health staff and teachers who now are being actively involved in primary eye care, screening and referral of eye patients, and must be included in the definition of the eye care team.

## Focusing on our patients

The concept of teamwork has been perfected by successful manufacturing companies, where people with different skills come together to make a car or other product which is then marketed, sold, and supported with after-sales service. Teams in health care may face many additional challenges, including funding and wider health systems issues. We deal with human beings and their sight; therefore we should do our best to provide not just great service, but also to offer a positive experience to our patients. For many of them, there is no second chance, so we must ensure that a high quality service is delivered the first time. Teamwork can help by enhancing the efficiency and quality of our work, both of which are essential to improve the vision and quality of life of our patients.

